# Extracorporeal Cardiopulmonary Resuscitation for Cardiac Arrest and Myocardial Infarction: A Diagnosis Beyond the Obvious

**DOI:** 10.1016/j.acepjo.2025.100238

**Published:** 2025-08-11

**Authors:** Georges Abi Abdallah, Anne-Laure Gaultier, Anne Godier

**Affiliations:** 1 Department of Anaesthesiology and Critical Care, AP-HP, European Georges Pompidou Hospital, Paris, France; 2 Innovative Therapies in Haemostasis, INSERM, F-75006 Paris, Université Paris Cité, France; 3 Department of Radiology, AP-HP, European Georges Pompidou Hospital, Paris, France

**Keywords:** acute type A aortic dissection, misdiagnosis, extracorporeal cardiopulmonary resuscitation

## 1 Case Presentation

A 66-year-old woman with no prior medical history presented to the emergency department with acute chest pain and anterior ST-segment elevation on the electrocardiogram. She subsequently went into cardiac arrest. Despite optimal cardiopulmonary resuscitation (CPR), spontaneous circulation could not be restored. Transthoracic echocardiography (TTE) performed during CPR showed no pericardial effusion, no intimal flap of the ascending aorta, and no other reversible cause; thus, extracorporeal cardiopulmonary resuscitation (ECPR) was initiated, and femoro-femoral cannulation achieved adequate initial extracorporeal membrane oxygenation flow. Ultrasound-guided verification of cannula placement revealed an aortic flap in the abdominal aorta. A subsequent angio-computed tomography scan was favored over first-line coronary angiography (Figure).

**Figure:**
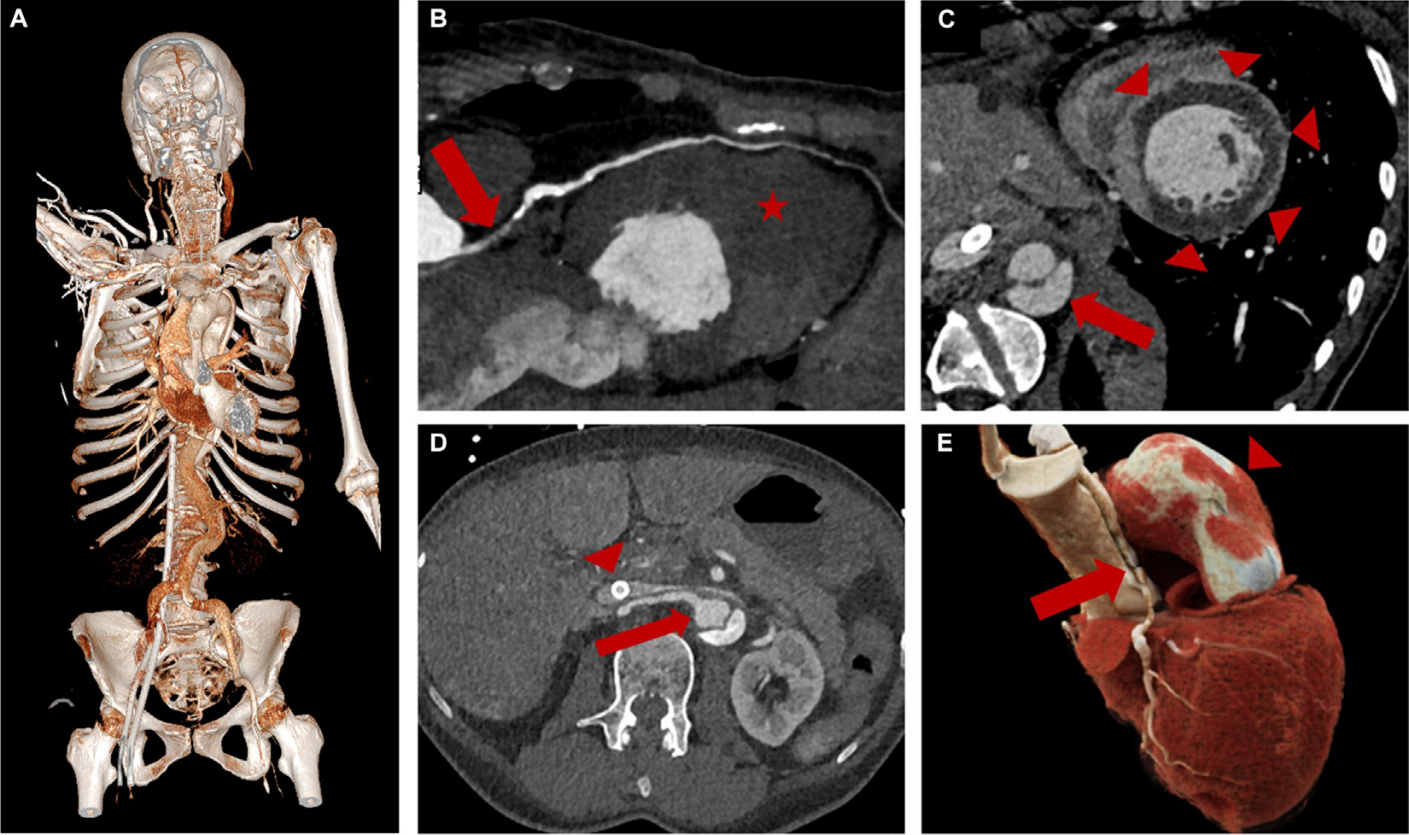
(A) Computed tomography images in three-dimensional rendered format showing the aortic dissection extending from the aortic root to the iliac bifurcation, with the venous cannula positioned in the inferior vena cava and the arterial cannula in the right iliac artery, located within the true lumen. (B) Dissection (arrow) of the left anterior descending artery along its entire length, resulting in a large myocardial infarction involving the entire left ventricle (star). (C) Short axis cardiac view showing a large myocardial infarction of the left ventricle (arrowheads) and dissected abdominal aorta (arrow). (D) Venous cannula located in the inferior vena cava (arrowhead), with the right renal artery arising from the true lumen (arrow) of the dissected abdominal aorta. (E) Left-sided view of a three-dimensional reconstruction (Siemens Healthineers Syngo.via) of the heart, ascending aorta and left anterior descending artery (LAD), showing enlarged diameter of the ascending aorta due to ATAAD (arrowhead) and stenotic LAD (arrow), caused by the extension of the ascending aorta dissection. 3D, three-dimensional; ATAAD, acute type A aortic dissection.

## 2 Diagnosis: Acute Type A Aortic Dissection with Coronary Involvement

Acute type A aortic dissection (ATAAD) is a life-threatening condition requiring prompt diagnosis and urgent surgical intervention. Despite significant advancements in surgical techniques and perioperative care, mortality remains high, above 20%.^1^ The wide variability in clinical presentations frequently leads to diagnostic challenges, which can delay surgical treatment or result in the administration of inappropriate treatments. In its most critical form, ATAAD can manifest as cardiac arrest, resulting from mechanisms such as hemorrhage due to aortic rupture, pericardial effusion, or ventricular infarction due to coronary involvement. The need for CPR further worsens the pre-existing dismal prognosis, and survival outcomes for ATAAD-related cardiac arrest remain highly variable in the literature, with reported rates ranging from 10% to 50%, with a clear trend toward improved outcomes in in-hospital cardiac arrest (IHCA).^2^ This case highlights the diagnostic challenges of ATAAD and the limitations of TTE, particularly in emergency settings. It underscores the importance of repeated imaging and continuous clinical reassessment. When ATAAD is suspected during resuscitation, transesophageal echocardiography should be preferred, as it offers superior sensitivity and allows real-time assessment without interrupting chest compressions.^3^ Finally, although ECPR is generally contraindicated in ATAAD due to the risk of aortic rupture and cannulating the false lumen, successful arterial cannulation of the true lumen in selected cases of IHCA with short CPR durations may facilitate vessel patency and organ perfusion as a bridge to surgery.^2^^,^^4^

## Funding and Support

By *JACEP Open* policy, all authors are required to disclose any and all commercial, financial, and other relationships in any way related to the subject of this article as per ICMJE conflict of interest guidelines (see www.icmje.org). The authors have stated that no such relationships exist.

## Conflict of Interest

All authors have affirmed they have no conflicts of interest to declare.
